# Retention Rates and the Associated Risk Factors of Turnover among Newly Hired Nurses at South Korean Hospitals: A Retrospective Cohort Study

**DOI:** 10.3390/ijerph181910013

**Published:** 2021-09-23

**Authors:** Yunmi Kim, Hyun-Young Kim

**Affiliations:** 1 Department of Nursing, College of Nursing, Eulji University, Seongnam-si 13135, Korea; kyunm@eulji.ac.kr; 2 Department of Nursing, College of Medical Sciences, Jeonju University, Jeonju-si 55069, Korea

**Keywords:** newly hired nurse, working period, turnover, hazard ratio

## Abstract

This retrospective cohort study analyzed the turnover rate and the risk factors of turnover among newly hired nurses at tertiary and secondary hospitals in South Korea. Using National Health Insurance Service data, this study created a cohort of 21,050 newly hired nurses across 304 hospitals in 2018, with a follow-up period of 18 months. Retention and turnover risk factors were analyzed at 6-month intervals. Differences in retention period according to hospitals’ organizational characteristics and nurses’ individual characteristics were analyzed using the chi-squared test. The likelihood of staying at work was analyzed using Kaplan–Meier survival curves with the log-rank test, and the hazard ratios of turnover at each retention period were analyzed using multilevel Cox proportional hazards analysis. The turnover rate of newly hired nurses within 1 year of employment was 26.4%, with 20.1% resigning within 6 months, and 6.3% resigning between 7 and 12 months. For all retention periods, turnover risk was associated with a higher bed-to-nurse ratio and older age. Higher standardized monthly income was associated with lower turnover between 13 and 18 months. Hospitals should develop nurse-retention strategies that consider risk factors for turnover. To reduce turnover, adequate nursing personnel should be maintained and fair compensation should be offered.

## 1. Introduction

### 1.1. Background

Clinical nurses account for about 45% of medical personnel at South Korean medical institutions [1], and they directly impact the quality of medical services, such as patient safety, as well as the financial aspects of hospital operations [2,3]. As the population of older adults increases and the global coronavirus disease 2019 (COVID-19) pandemic continues, experienced nurses who can take on challenging nursing work are more crucial than ever, and a strong labor force of nurses is increasingly needed [4,5,6]. Many countries, including South Korea (hereafter, Korea), suffer from shortages of nurses [4,7]; thus, Korea is expected to require a labor force of approximately 280,000 nurses by 2030—about 150,000 more nurses than were in practice in 2015 [8]. As of 2019, the number of practicing nurses was 193,043, which is fewer than half of the 414,983 registered nurses (RNs) in Korea [1], and the proportion of clinical nurses (including RNs and nurse aides) for every 1000 people was 7.2 in 2018, which is 1.7 fewer than the Organization for Economic Co-operation and Development (OECD) average of 8.9 [9]. In most OECD nations, nurse aides accounted for around 20% of the total clinical nursing staff, whereas in Korea, the proportion of RNs per 1000 people was only 3.78 (51%) in 2018 [10], which is less than half the level of the OECD average.

Although the Korean government has gradually increased the entrance quota for nursing colleges since 2008 to train more nurses and address the shortage of clinical nurses, this increase has had a minimal effect on local small- and mid-sized hospitals, where the situation has not improved and, in some instances, has deteriorated [11]. Since March 2018, an additional measure has been in place to improve nurses’ working environment and compensation [8]. The main reason a shortage of nurses persists in Korea, despite the measures which have been taken, is the high turnover rate of nurses. The average employment period of Korean nurses is 5.4 years, and the cumulative turnover rate within 5 years of employment was found to be 49.7% in a study conducted between 2012 and 2016, which suggests that skilled nurses did not stay at their jobs for a long-term period [8,12]. Moreover, the turnover hazard ratio of new nurses with less than 1 year of experience has been found to be 111% higher than that of nurses with 5 or more years of experience, thus explaining why the shortage of nurses continues even with more nursing graduates [12].

Given these circumstances, the main factors that lead to turnover among nurses in Korea must be identified, especially among new nurses, for whom the rate of turnover is particularly high. According to the Korean clinical ladder system model, which was developed using Benner’s “From Novice to Expert” theory, the present study assumed that a novice nurse becomes an advanced beginner after 1 year and a competent nurse after 3 years [13]. Based on this assumption, turnover around the time when a nurse is about to reach the competent level is damaging to both the individual and the organization, resulting in additional costs for the organization to hire and train a new nurse, lowering nursing quality, and posing challenges for the nurse as they adjust to a new organization or seek work as an experienced nurse [14,15]. A study on a critical care nursing surge model during the COVID-19 pandemic found that nurses with a minimum of 2 years of experience were critical for maximizing nursing competency and ensuring patient safety [5].

### 1.2. Research Purpose

The present study is meaningful in that it analyzed actual turnover data on newly hired nurses as opposed to data on nurses’ intentions to leave their jobs within 18 months of employment [16,17]. It is hoped that the results of this study will provide a foundation for developing effective strategies to reduce turnover among newly hired nurses by suggesting outcomes based on analyses of data at both the individual and organizational level to reflect the characteristics of clustered data that include more than one nurse per medical institution [18].

## 2. Materials and Methods

### 2.1. Design

This was a retrospective cohort study that conducted a secondary analysis of National Health Insurance Service (NHIS) data from January 2018 to July 2020 to examine the retention periods, associations between individual and organizational factors, and the actual turnover of newly hired nurses.

### 2.2. Setting and Data Collection

Cohort data from 21,050 nurses were used in this study from NHIS data submitted by 43 tertiary hospitals and 261 secondary hospitals, accounting for 86.1% of these hospitals in Korea. A cohort was built by connecting 2018 data on newly hired nurses and data from 2019 and July 2020 on employment among nurses. Follow-up was conducted over an 18-month period, at which the employment status of all newly hired nurses in the cohort could be confirmed. In other words, the data were followed until July 2020, which was when the employment status of nurses who started working in December 2018 could be confirmed.

### 2.3. Variables

The dependent variable of this study was turnover among nurses. Turnover means that nurses left the hospitals where they were working and either moved to another clinical setting or decided to stop working as clinical nurses. All turnover events that took place during the follow-up period were confirmed with medical institutions by the researchers, and nurses with no turnover events were categorized as having been retained. Retention periods were categorized into <7 months, ≥7 and <13 months, ≥13 and <19 months, and ≥19 months, based on earlier studies on the experiences of student nurses as they transitioned to become newly hired RNs [17,19,20]. The retention period was calculated from the date of employment to the date of resignation for nurses who experienced turnover events during the follow-up period, and nurses who did not experience a turnover event within 18 months were categorized as having a retention period of ≥19 months.

Independent variables were decided separately at both the organizational level and the individual level, based on an earlier study [14]. The organizational variables of hospitals that were selected were hospital location, hospital ownership, bed-to-nurse ratio, and standardized monthly income [12,17,21,22], whereas gender and age were selected to be the individual variables of nurses [12,22]. Sub-variables for hospital location were Seoul, metropolitan cities, and medium-sized and small cities, and the sub-variables for hospital ownership were public sector, educational foundation, medical or other corporation, and for-profit medical institutions. The bed-to-nurse ratio was the mean number of beds divided by the mean number of full-time equivalent nurses as of the second quarter of 2018 at general wards, and was divided into 4 categories (<2.5, ≥2.5 to <3.5, ≥3.5 to <4.5, or ≥4.5) [12,23]. Standardized monthly income (average monthly compensation) was defined as the standardized income at each medical institution used to determine the health care premiums of nurses. It was determined using the amounts reported to the NHIS. The amount was estimated in two steps to obtain an average amount that accurately reflected actual income. During the first step, the average monthly income and standard deviation (SD) of salaries from December 2018 for each medical institution were calculated for female nurses 24 years of age or younger who started working before June 2018, considering that many hospitals offer 100% of salaries to newly hired nurses after a probation period of 3–6 months. During the second step, the standardized monthly income was determined using the new average after excluding the 1% outliers outside the mean ± SD [24]. Incomes were categorized into 4 groups: <2500, 2500–2999, 3000–3499, and ≥3500 (units: Korean Won (KRW) 1000); corresponding to approximately USD <2100, USD 2100–2500, USD 2500–3000, and USD >3000).

### 2.4. Data Analysis

The differences between the retention periods of newly hired nurses according to organizational and individual characteristics were analyzed using the chi-squared test. The likelihood of remaining at work by organizational and individual characteristics was analyzed using Kaplan–Meier survival curves with the log-rank test. The turnover risk factors according to organizational and individual characteristics in all retention period groups were analyzed using multilevel Cox proportional hazards analysis, incorporating cluster-specific random effects. The data used in this study had a two-level data structure in the form of nurses nested within hospitals; therefore, we used a multilevel survival model to perform valid inferences on the effects of both individual characteristics and cluster characteristics on the risk of an outcome’s occurrence [18]. The significance level was set at *p* < 0.05 and the confidence interval (CI) was set at 95%. For all analyses, SAS 9.4 was used (SAS Institute, Cary, NC, USA).

## 3. Results

### 3.1. The Characteristics of Hospitals and Nurses

It was found that 54.3% of the hospitals included in this study were located in medium-sized or small cities, and that 44.6% of the nurses included in this study worked at these hospitals. Ownership by medical or other corporations accounted for 42.1% of hospitals with which 34.5% of the nurses were affiliated. Educational foundations owned only 20.1% of hospitals, but they hired the largest number of nurses (36.9%). In total, 41.1% of hospitals had a bed-to-nurse ratio of ≥2.5 to <3.5; however, the largest group of nurses (46.6%) worked at hospitals with bed-to-nurse ratios of <2.5. Most hospitals (43.1%) and nurses (42.4%) had a standardized monthly income of 2500–2999 (units: KRW 1000). An absolute majority (90.7%) of the nurses were women, and 48.9% were 24 years of age or younger (Table 1).

### 3.2. Nurse Retention Period According to Organizational and Individual Characteristics

Among the 21,050 newly hired nurses, 4234 (20.1%) were employed for <7 months, 1332 (6.3%) for ≥7 to <13 months, 2340 (11.1%) for ≥13 to <19 months, and 13,144 (62.4%) for ≥19 months. In total, 5566 nurses were employed for less than 1 year, indicating a 26.4% turnover rate (Table 2).

There were significant differences in the rate of nurses with retention periods of ≥19 months depending on whether the hospital was located in Seoul (66.5%), a metropolitan city (61.0%), or a medium-sized or small city (61.3%) (χ^2^ = 48.19, *p* < 0.001). Differences in the retention periods of newly hired nurses were also statistically significant according to hospital ownership, with public sector hospitals having the highest proportion of retention periods of ≥19 months (70.1%), followed by hospitals owned by educational foundations (62.9%), hospitals owned by medical or other corporations (59.3%), and for-profit medical institutions (55.9%) (χ^2^ = 184.16, *p* < 0.001). A retention rate of ≥19 months was also the highest (68.4%) among nurses who worked at institutions with a bed-to-nurse ratio of <2.5, and was the lowest (53.5%) at institutions with a bed-to-nurse ratio of ≥4.5, showing a significant gap (χ^2^ = 308.56, *p* < 0.001). The retention rate was ≥19 months at 73.3% of hospitals that offered newly hired nurses a standardized monthly income of ≥3500 (units: KRW 1000), 62.1% of hospitals that offered a monthly income of 3000–3499, and 60.5% of hospitals that offered <3000 KRW (χ^2^ = 170.25, *p* < 0.001) (Table 2).

The percentage of new male nurses who stayed in their roles for ≥19 months was 57.6%, which was significantly lower than that of their female peers (62.9%) (χ^2^ = 22.53, *p* < 0.001). The 19-month retention rate was the highest among nurses who were 24 years of age and younger, at 67.5%, with a statistically significant decreasing trend as age increased (χ^2^ = 313.20, *p* < 0.001) (Table 3).

### 3.3. Likelihood of Remaining at Work by Organizational and Individual Characteristics

**Figure 1:**
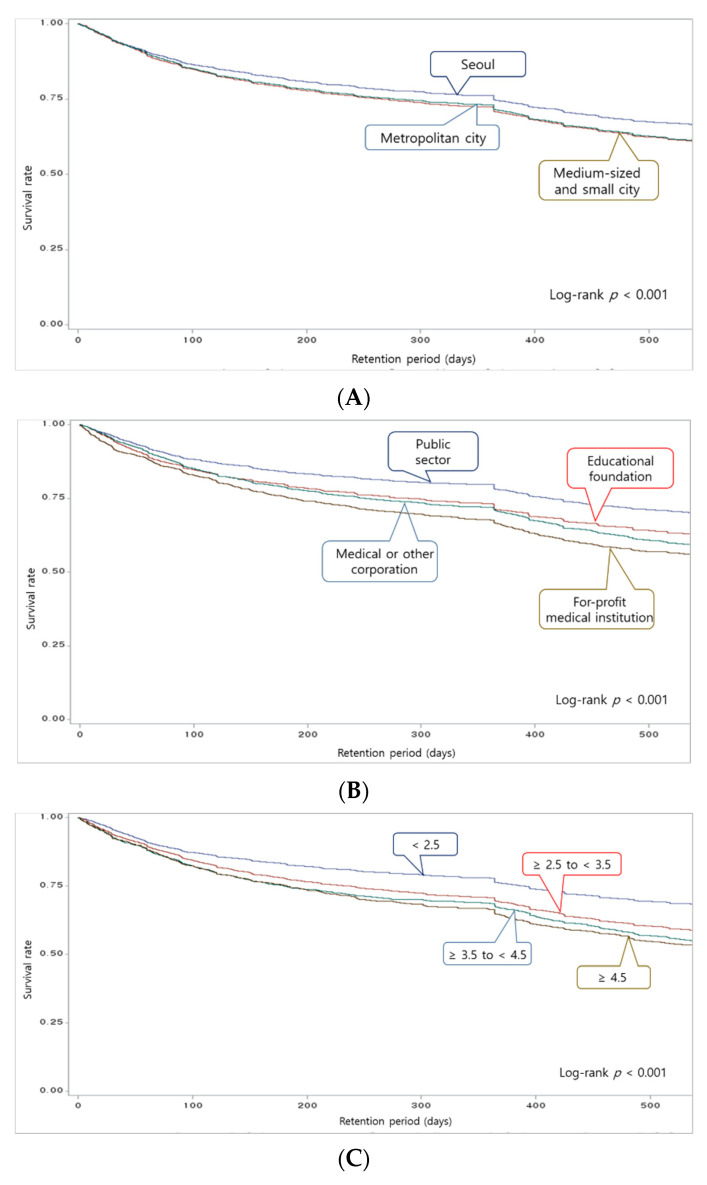

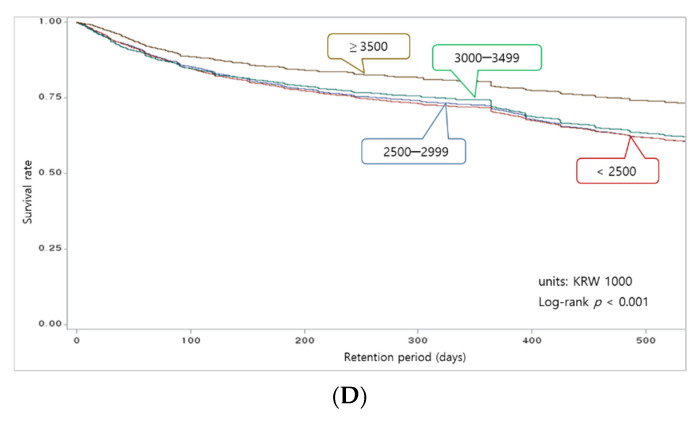
Survival curves by hospitals’ characteristics (**A**) Comparison of the likelihood of remaining at work by hospital location. (**B**) Comparison of the likelihood of remaining at work by hospital ownership. (**C**) Comparison of the likelihood of remaining at work by bed-to-nurse ratio. (**D**) Comparison of the likelihood of remaining at work by standardized monthly income. KRW, Korean Won.

The likelihood of remaining at work according to the organizational characteristics of medical institutions was generally higher at hospitals located in Seoul than at hospitals in other regions (*p* < 0.001). The likelihood of remaining at work was highest at hospitals owned by the public sector and the lowest at for-profit medical institutions (*p* < 0.001). It was also the highest when the bed-to-nurse ratio was <2.5 (*p* < 0.001). The likelihood of remaining at work was also the highest when the monthly income of nurses was ≥3500 (units: KRW 1000; *p* < 0.001) (Figure 1).

**Figure 2:**
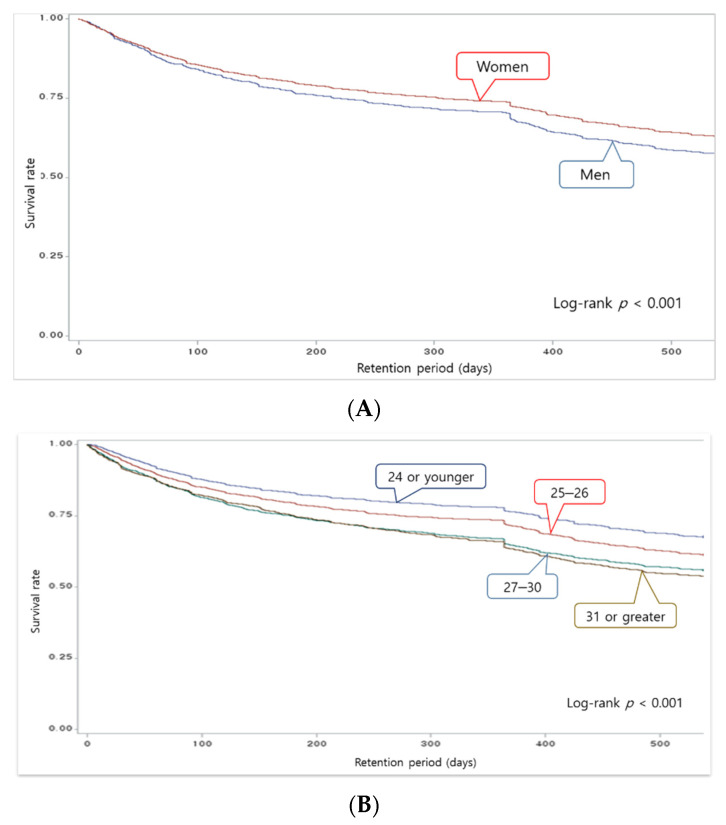
Survival curves by nurses’ characteristics. (**A**) Comparison of the likelihood of remaining at work by gender. (**B**) Comparison of the likelihood of remaining at work by age.

Male nurses had a lower likelihood of remaining at work than females (*p* < 0.001), and the rate decreased as nurses’ ages increased (*p* < 0.001) (Figure 2). All survival curves according to the characteristics of hospitals and nurses showed a significant drop between 300 and 400 days of employment.

### 3.4. Risk Factors for Turnover by Retention Period

The main risk factors for turnover among new nurses within 6 months of employment were age, hospital ownership, and the bed-to-nurse ratio. As the age groups of nurses increased to 25–26, 27–30, and 31 or older, the turnover risk increased by 22%, 41%, and 31%, respectively, compared to nurses who were 24 or younger. Public sector hospitals had a 22% lower turnover risk than for-profit hospitals. The turnover risk decreased by 29% as the bed-to-nurse ratio decreased from ≥4.5 to <2.5.

The main turnover risk factors for nurses who remained employed for ≥7 and <13 months were age, hospital ownership, and the bed-to-nurse ratio. Compared to nurses who were 24 years old and younger, nurses who were in the 25–26, 27–30, and 31 or older age groups had a 26%, 65%, and 78% higher risk of turnover, respectively. Public sector hospitals had a 31% lower turnover risk than for-profit medical institutions. Turnover rates decreased by 27% when the bed-to-nurse ratio improved from ≥4.5 beds per nurse to ≥3.5 to <4.5 beds per nurse, and by 34% when it improved to <2.5 beds per nurse.

The main turnover risk factors for nurses who remained employed for ≥13 and <19 months were age, the bed-to-nurse ratio, and standardized monthly income. Compared to nurses who were 24 years old or younger, turnover rates were higher by 22% and 16% in the 25–26 and 31 or older age groups, respectively. Turnover rates decreased by 29% when the bed-to-nurse ratio changed from ≥4.5 beds per nurse to <2.5. In addition, the turnover risk decreased by 38% when the standardized monthly income increased from <2500 to ≥3500 (units: KRW 1000) (Table 4).

## 4. Discussion

The purpose of the present study was to examine the retention period, likelihood of remaining at work, and other factors associated with turnover among newly hired nurses at hospitals in Korea. The study found a turnover rate of 26.4% within 1 year of employment among newly hired nurses, and the likelihood of remaining at work was found to decrease sharply between 300 and 400 days of retention in all survival curves. The analysis of risk factors associated with turnover by retention period found that the bed-to-nurse ratio of an organization and the age of an individual nurse were the characteristics that had statistically significant impacts on turnover within all retention periods. In addition, offering a monthly income of ≥3500 (units: KRW 1000) lowered turnover rates between 13 and 18 months of employment.

In total, 20.1% of nurses in this study left their jobs at <7 months of employment, 6.3% left between 7 and 12 months, 11.1% left between 13 and 18 months, and 62.4% did not experience a turnover event within the 18-month follow-up period. The 26.4% turnover rate of new nurses within 1 year of service between 2018 and 2019 found in the present study was higher than the 22% turnover rate found in another study of new nurses at secondary and tertiary hospitals between 2012 and 2016 that used the same criteria [12]. It was also higher than the 17.7% turnover rate of new nurses within 1 year of service found in another study that analyzed the actual turnover rate between 2006 and 2008 using different datasets [25]. This result suggests that the turnover rate of new nurses has continued to rise even as the Korean government has implemented policies to increase the labor force of nurses and reduce turnover [8,11]. In comparison, Korea’s closest neighbor, Japan, has maintained a stable turnover rate of 11% or lower among nurses for more than a decade; the overall turnover rate of nurses in Japan was 10.7% in 2019. Moreover, the turnover rate of Japanese nurses within 1 year of employment was 7.8% in 2019, which was even lower than the overall turnover rate, and vastly different from the situation in Korea [26]. In the future, a detailed analysis of the factors associated with turnover among new nurses in Korea would be helpful for developing a deeper understanding of this issue.

The present study also found that the turnover rate of newly hired nurses differed between 6-month intervals and increased as nurses marked their 1-year anniversaries of employment. In an earlier study on the turnover intentions of newly hired nurses, turnover intentions increased significantly at 5 months of employment compared to 2 months, and the difference between turnover intentions at less than 6 months of employment and at 6 months of employment or above was also statistically significant [16,17]. Given these outcomes, it can be inferred that actual turnover and turnover intentions change within relatively short periods of time, such as intervals of 3–6 months, although a direct comparison was impossible due to a lack of studies that examined actual turnover rather than turnover intentions. Changes in short cycles should thus be considered when implementing strategies to prevent turnover among new nurses.

The Kaplan–Meier survival curve of the present study indicated a sharp drop in the likelihood of remaining at work across all graphs between 300 and 400 days of employment. This is likely due to the common practice of requiring nurses to have at least 1 year of experience to be eligible for more advanced roles at medical institutions. An earlier study confirmed that 76.1% of new nurses who left their jobs were employed by a different institution within 1 year of turnover [27]. This study found that there was more likely to be a turnover of newly hired nurses who were 31 years of age or older before they reached 6 months of employment (45%) and between 7 and 12 months of employment (78%) than nurses who were 24 years of age and younger. These findings suggest that there may be a vicious cycle in which newly hired nurses with prior nursing experience have a high turnover risk after already having left a previous nursing job for better working conditions. This is also supported by a preceding study that examined turnover among new nurses and more experienced nurses, and found that the average age of new nurses was 23.19 years old and 34.54 years old for experienced nurses [28]. These findings suggest that a majority of the nurses in the present study who were 31 years of age or older may already have had professional nursing experience. This underscores the importance of devising strategies to prevent experienced nurses from leaving their roles in search of better working conditions after around 1 year of employment.

The staffing level is a factor highly associated with patient outcomes and burnout among nurses [4,14,21]. In the present study, the bed-to-nurse ratio consistently appeared to be a risk factor for turnover across all retention periods grouped into 6-month intervals. This aligns with the results of earlier studies in Korea [12,22]. Hospitals with a bed-to-nurse ratio of 3.5 or higher are technically in violation of the 5-to-2 patient-to-nurse ratio stipulated by Korean medical law [29], which means that 38.5% of the hospitals in this study did not comply with the minimum legal staffing requirement. In fact, the nurse-to-bed ratio according to head count data released by the OECD in 2018 put Korea among the lowest level at 0.38, which was significantly lower than Denmark’s score at 2.95, Canada’s at 2.48, and Chile’s at 0.46 [30]. The most crucial task for maintaining a higher nurse retention rate would be to develop policies to improve poor work environments, which are highly associated with heavy workloads caused by inadequate staffing and high turnover [4]. The findings of the present study and previous studies suggest that inadequate staffing at medical institutions can result in heavy workloads for nurses, leading to a higher turnover rate among nurses, which, in turn, aggravates the intensity of the remaining nurses’ workloads.

Ownership of hospitals by the public sector had a proven impact on lowering the risk of turnover within 1 year of employment, which can be explained by the monopsonic character of medical institutions that hire nurses. Monopsony has long been considered a cause of the persistent nursing shortage because it allows medical institutions to hire staff at a low cost [31,32]. In the labor market of nurses in Korea, major cities such as Seoul, where most of the nation’s large-scale hospitals with high numbers of beds are concentrated, can maintain the labor market of nurses according to the principle of supply and demand, whereas for-profit hospitals in small cities could maintain lower wages due to the monopsony [24]. In 2008, the Korean government began to implement a policy to increase the supply of nurses by accepting more nursing students into nursing schools; however, the policy actually backfired, because medical institutions no longer needed to raise nurses’ pay or improve working conditions to attract more nurses to work at their institutions. A study that compared the initial salaries of clinical nurses at public sector hospitals in nine Asian countries after adjusting for purchasing power parity confirmed that the initial salary of Korean clinical nurses in 2006 was the second highest, after Hong Kong, but dropped to the fifth highest in 2016, after Hong Kong, Japan, and other countries [33]. In markets where monopsony exists, government intervention is necessary to maintain balance in the market, which also explains why nurses’ incomes were 7.3% higher at publicly owned hospitals than at privately owned hospitals in the present study, ultimately lowering the turnover risk [24]. Higher standardized monthly income would contribute to lowering the turnover risk after 1 year of employment (≥13 and <19 months), which is supported by the findings of the present study in which hospitals that paid a standardized monthly income of KRW 3.5 million had a 38% lower turnover risk than those that paid their nurses less than KRW 2.5 million per month. Countries such as the United Kingdom, Australia, and Germany have standard salary guidelines that are unrelated to hospital ownership or the number of beds, but rather are based on the level of professional experience of nurses [7,24]. In other words, given that increased pay reduces both turnover intentions and actual turnover among nurses, a wage policy that corresponds to the degree of professional experience of nurses to attract non-working nurses back to the labor force would help to address the nursing shortage [6,31].

A bundled approach that ensures adequate staffing at medical institutions, raises the salaries of nurses, and improves the work culture at medical institutions will likely be the most effective approach [4] for encouraging newly hired nurses to remain in their jobs for 1 year or more, develop expertise, and ensure patient safety, enhanced nursing quality, and protect Korean citizens’ right to a healthy life [5,13]. To this end, it is necessary to strengthen medical law in Korea regarding staffing levels by referring to the highly effective minimum nurse staffing level policy that was implemented in 15 U.S. states in hospitals with low levels of nurse recruitment [34]. Japan also introduced a new nurse training system in 2010 at the national level, in which the government manages and subsidizes the cost of support for new nurses and for preceptors who offer education, which helped maintain Japan’s turnover rate of new nurses within 1 year of employment at 8% or less [26]. Based on the outcomes of the present study and the new nurse training system of Japan, it is necessary to introduce a system in Korea wherein standardized education for a sufficient period is available with financial support from the government rather than entrusting the training of newly hired nurses to individual medical institutions. In addition, strategies must be implemented to lower the turnover rate of nurses such as creating a wage system based on the retention periods used in this study. When nurses reach 1 year of employment, their standardized monthly income should increase accordingly, thus also raising retention rates at medical institutions. In other words, steps must be taken to ensure that new nurses who are at transitional stages in their professional development and on their way to becoming competent nurses do not have to move between institutions seeking better working conditions, resulting in losses to organizations and individuals. Thus, standardized salary guidelines and wage policies should be implemented based on nurses’ experience levels rather than hospital ownership [24,31].

Despite these meaningful findings, the present study also has limitations. Although it was presumed that newly hired nurses from higher age groups in the present study were likely experienced nurses [28], this could not be confirmed because the datasets used in the study did not include data on the year of license acquisition and turnover; thus, it was not possible to differentiate between new graduates and experienced workers. Furthermore, we were not able to include job characteristics related to turnover, such as shift work and working hours, as variables [35]. Finally, the dataset in the present study contained data from the initial period of the COVID-19 pandemic (January–July 2020). Therefore, we could not evaluate whether nurses’ turnover has changed in response to COVID-19. Further research on COVID-19 and nurses’ turnover is necessary. However, it can still be inferred from the results of the present study that the risk of turnover among experienced nurses is increasing, and that active intervention is needed at the 1-year mark to prevent turnover.

## 5. Conclusions

The present study used cohort data within an 18-month follow-up period to analyze actual turnover among newly hired nurses. The validity of the study was strengthened by the multilevel analysis of data at the individual and organizational levels. The results showed that the rate of turnover among newly hired nurses within 1 year of employment was 26.4%, that a lower bed-to-nurse ratio corresponded to a lower turnover risk across all retention periods, and that the turnover risk decreased after 1 year of employment due to higher standardized monthly income. Improving the two working conditions (bed-to-nurse ratio and income) is crucial for maintaining higher retention rates of nurses. Government-level intervention is required based on the successful strategies implemented in other countries. Turnover rates and risk factors also varied according to the employment period. Prospective studies should be conducted in the future to determine the effects of strategies for promoting job retention and to understand the factors that affect turnover among nurses.

## Figures and Tables

**Table 1:** Organizational and individual characteristics.

Category	Hospital (*n* = 304)	Nurse (*n* = 21,050)
Organization		
Hospital location		
Seoul	52 (17.1)	5096 (24.2)
Metropolitan city	87 (28.6)	6564 (31.2)
Medium-sized and small city	165 (54.3)	9390 (44.6)
Hospital ownership		
Public sector	63 (20.7)	4132 (19.6)
Educational foundation	61 (20.1)	7758 (36.9)
Medical or other corporation	128 (42.1)	7263 (34.5)
For-profit medical institution	52 (17.1)	1897 (9.0)
Bed-to-nurse ratio		
<2.5	62 (20.4)	9817 (46.6)
≥2.5 to <3.5	125 (41.1)	7643 (36.3)
≥3.5 to <4.5	49 (16.1)	1754 (8.3)
≥4.5	68 (22.4)	1836 (8.7)
Standardized monthly income (KRW 1000)		
<2500	112 (36.8)	5297 (25.2)
2500–2999	131 (43.1)	8915 (42.4)
3000–3499	45 (14.8)	4144 (19.7)
≥3500	16 (5.3)	2694 (12.8)
Individual nurse		
Gender	-	
Men	-	1968 (9.3)
Women	-	19,082 (90.7)
Age (years)		
≤24	-	10,292 (48.9)
25–26	-	4560 (21.7)
27–30	-	3266 (15.5)
≥31	-	2932 (13.9)

Values are presented as *n* (%); KRW, Korean Won.

**Table 2:** Differences between the retention periods of newly hired nurses according to organizational characteristics (*n* = 21,050).

Category	<7 Months	≥7 to <13 Months	≥13 to <19 Months	≥19 Months	χ^2^ (*p*)
Number	4234 (20.1)	1332 (6.3)	2340 (11.1)	13,144 (62.4)	-
Hospital location					
Seoul	925 (18.2)	291 (5.7)	493 (9.7)	3387 (66.5)	48.19 <0.001
Metropolitan city	1379 (21.0)	436 (6.6)	744 (11.3)	4005 (61.0)	
Medium-sized and small city	1930 (20.6)	605 (6.4)	1103 (11.8)	5752 (61.3)	
Hospital ownership					
Public sector	658 (15.9)	182 (4.4)	396 (9.6)	2896 (70.1)	184.16 <0.001
Educational foundation	1572 (20.3)	504 (6.5)	803 (10.4)	4879 (62.9)	
Medical or other corporation	1541 (21.2)	497 (6.8)	917 (12.6)	4308 (59.3)	
For-profit medical institution	463 (24.4)	149 (7.9)	224 (11.8)	1061 (55.9)	
Bed-to-nurse ratio					
<2.5	1647 (16.8)	518 (5.3)	942 (9.6)	6710 (68.4)	308.56 <0.001
≥2.5 to <3.5	1694 (22.2)	542 (7.1)	917 (12.0)	4490 (58.8)	
≥3.5 to <4.5	440 (25.1)	109 (6.2)	243 (13.9)	962 (54.9)	
≥4.5	453 (24.7)	163 (8.9)	238 (13.0)	982 (53.5)	
Standardized monthly income (1000 KRW)					
<2500	1104 (20.8)	353 (6.7)	635 (12.0)	3205 (60.5)	170.25 <0.001
2500–2999	1903 (21.4)	613 (6.9)	1009 (11.3)	5390 (60.5)	
3000–3499	828 (20.0)	238 (5.7)	504 (12.2)	2574 (62.1)	
≥3500	399 (14.8)	128 (4.8)	192 (7.1)	1975 (73.3)	

Values are presented as *n* (%); KRW, Korean Won.

**Table 3:** Differences between the retention periods of newly hired nurses according to general characteristics (*n* = 21,050).

Category	<7 Months	≥7 and <13 Months	≥13 and <19 Months	≥19 Months	χ^2^ (*p*)
Number	4234 (20.1)	1332 (6.3)	2340 (11.1)	13,144 (62.4)	
Gender					
Men	450 (22.9)	131 (6.7)	253 (12.9)	1134 (57.6)	22.53 <0.001
Women	3784 (19.8)	1201 (6.3)	2087 (10.9)	12,010 (62.9)	
Age (years)					
≤24	1746 (17.0)	532 (5.2)	1067 (10.4)	6947 (67.5)	313.20 <0.001
25–26	935 (20.5)	274 (6.0)	554 (12.2)	2797 (61.3)	
27–30	822 (25.2)	257 (7.9)	360 (11.0)	1827 (55.9)	
≥31	731 (24.9)	269 (9.2)	359 (12.2)	1573 (53.7)	

Values are presented as *n* (%).

**Table 4:** Characteristics of hospitals and nurses as risk factors of turnover among newly hired nurses (*n* = 21,050).

Category	Total		<7 Months		≥7 to <13 Months		≥13 to <19 Months	
	HR (95% CI)	*p*	HR (95% CI)	*p*	HR (95% CI)	*p*	HR (95% CI)	*p*
Gender (ref: women)								
Men	1.04 (0.96–1.12)	0.33	1.02 (0.92–1.13)	0.74	0.94 (0.78–1.15)	0.56	1.14 (0.99–1.32)	0.07
Age (years) (ref: ≤24)								
25–26	1.22 (1.15–1.30)	<0.001	1.22 (1.12–1.33)	<0.001	1.26 (1.08–1.47)	0.003	1.22 (1.09–1.36)	0.001
27–30	1.34 (1.26–1.44)	<0.001	1.41 (1.29–1.54)	<0.001	1.65 (1.41–1.94)	<0.001	1.10 (0.97–1.25)	0.14
≥31	1.32 (1.23–1.41)	<0.001	1.31 (1.19–1.44)	<0.001	1.78 (1.52–2.09)	<0.001	1.16 (1.02–1.32)	0.028
Hospital location (ref: medium-sized and small city)								
Seoul	1.08 (0.94–1.24)	0.26	1.12 (0.95–1.33)	0.19	1.05 (0.86–1.28)	0.64	1.00 (0.84–1.20)	0.96
Metropolitan city	1.08 (0.97–1.21)	0.16	1.09 (0.95–1.25)	0.21	1.10 (0.93–1.30)	0.26	1.03 (0.89–1.19)	0.70
Hospital ownership (ref: for-profit medical institution)								
Public sector	0.79 (0.67–0.94)	0.007	0.78 (0.63–0.97)	0.023	0.69 (0.52–0.91)	0.008	0.83 (0.66–1.05)	0.11
Educational foundation	1.04 (0.88–1.23)	0.67	1.02 (0.83–1.25)	0.88	1.10 (0.85–1.43)	0.45	1.02 (0.81–1.28)	0.86
Medical or other corp.	1.00 (0.86–1.15)	0.95	0.95 (0.80–1.13)	0.54	0.95 (0.76–1.19)	0.66	1.13 (0.93–1.38)	0.21
Bed-to-nurse ratio (ref: ≥4.5)								
<2.5	0.69 (0.58–0.82)	<0.001	0.71 (0.57–0.87)	0.001	0.66 (0.51–0.86)	0.002	0.71 (0.56–0.89)	0.003
≥2.5 to <3.5	0.86 (0.74–0.99)	0.034	0.86 (0.72–1.03)	0.11	0.85 (0.68–1.07)	0.16	0.89 (0.72–1.08)	0.23
≥3.5 to <4.4	0.93 (0.79–1.10)	0.40	0.99 (0.80–1.22)	0.93	0.73 (0.55–0.97)	0.028	1.01 (0.80–1.27)	0.95
Standardized income (1000 KRW/month) (ref: <2500)								
2500–2999	1.06 (0.95–1.19)	0.26	1.09 (0.95–1.24)	0.24	1.12 (0.95–1.32)	0.19	0.97 (0.84–1.12)	0.68
3000–3499	1.03 (0.88–1.19)	0.74	1.05 (0.87–1.26)	0.61	0.93 (0.74–1.16)	0.50	1.05 (0.87–1.27)	0.61
≥3500	0.85 (0.68–1.06)	0.16	0.81 (0.56–1.19)	0.29	0.89 (0.65–1.21)	0.44	0.62 (0.47–0.83)	0.001

HR, hazard ratio; CI, confidence interval; KRW, Korean Won.

## Data Availability

The cohort datasets were collected from National Health Insurance Service (NHIS) data on nurses’ hiring information submitted by hospitals. Although the dataset does not contain personally identifiable information, it is not publicly available because it is only available to persons authorized by the NHIS. The first author obtained approval to use NHIS data (NHIS-2021-1-527), and the extracted data were analyzed in a data laboratory designated for that purpose.
